# Annual Meeting Minnesota Dental Association

**Published:** 1874-10

**Authors:** 


### ANNUAL MEETING OF THE MINNESOTA DENTAL ASSOCIATION.

The Minnesota State Dental Association convened at 10 o'clock, a. m., at Theopold's Hall, in the town of Faribault, on Wednesday. Dr. Bowman, of Minneapolis, in the chair. The attendance of gentlemen from all parts of the state was good, considering the intense heat of the weather. There were representatives from almost all parts of the country, and particularly from the lower part of the State. The design was to so locate the annual convention of the Association, as to make the gathering as near the centre of the thickly settled portion of the State as possible. The members of the profession throughout the State are in number about one hundred, as at present on the rolls.

After the admission of several new members, signing of the Constitution and By-Laws was through with, the regular order of exercises was at once entered upon, and several hours of both Wednesday and Thursday were consumed in listening to the addresses and essays upon the subjects of "Sensitive Dentine" "Separation of the teeth" "Alveolar Abscess, Cause and Cure" and "Reflex Action of the Fifth Pair of Nerves" "Apthous Diseases of the Mouth" etc., etc., and the discussions thereon by all of those present. The essays most prominent among the number read were those by Drs. C. E. Miller, of Rochester; Hurd, of Faribault; DeMontreville, of St. Paul; Williamson, of Red Wing, and others. They interested the attention of the audience by the originality therein presented, showing that we have a body of special investigators in our midst, that in their specialty are eminently practical and very utilitarian in their views.

Among other questions discussed was the subject of special legislation to regulate the practice of the profession in our State, which, however was finally passed over and laid on the table till the next annual meeting, so that full time might be given to hear of the workings of legislation in the states, Pennsylvania, Ohio, New York and other states which have found laws necessary as a protection for the public to guard them against the malpractice of charlatans and quacks, that through the tempting bait of cheap work so sucessfully delude the public.

Also another question came before the Association, which was the necessiity of so localizing the annual gathering to some central place that the nucleus of a dental college and museum of morbid specimens of dental mechanism might be gradually gathered together, as an earnest of the progress of the profession, and around which the State Association can cast its protection. St. Paul, as the great commercial center after full discussion, was selected as the point where in future the annual conventions will assemble, and where the Cabinet of Dental Specimens it is to be hoped in time will rival those of our eastern brethren. The honor of the first contribution of morbid anomalies must be awarded to Dr. Cole, o fOwatonna, and also his beautiful specimens of celluloid work, which were duly placed in the cabinet, which for the present (or until a constitutional officer is provided) will be in the charge of the Treasurer.

Among the many articles of dental mechanism we noted prominently the very great hold that the new substance called Celluloid has attained in the affections of the public, and particularly some half dozen pieces in the various stages of manufacture, by one of the profession from St. Paul, which we are sure will be very difficult to improve upon, and it was very unanimously the opinion of the members present, that such work as there was exhibited, if it should be found as durable as the old fashioned rubber or vulcanite work, will very soon supersede that and all other kinds. It approached nearer to perfection than anything we ever saw, was easy of manipulation and in price about the same as rubber and subject to the arbitrary dictum of no patentees, royalty or vexatious suits of infringement; and in short, we think the exhibition of so many pieces of new celluloid work, some of which had been worn for three years and upward was decidedly *the success* of the annual gathering, as showing to the mass of those present what had been accomplished by the advanced members of the profession.

By a full vote of those present, the annual time of meeting was changed, and for the future the Association will assemble on the last Wednesday of May, so as to avoid the intense heat of the season such as we are now experiencing, and which to endure in a packed convention is no easy trial.

The choice of the Association for officers for the coming year was unanimously in favor of Dr. DeMontreville, of St. Paul, as President; Dr. Williamson, of Red Wing, Vice President; Dr. J. H. Bryant, of St. Paul, Secretary; and the present incumbent, Dr. S. Beecher, of St Paul, Treasurer. After the regular calendar of business was gone through with the Association adjourned to the office of Dr. Hurd, of Faribault, when clinics were held at which Dr. Griswold, of Minneapolis (a late acquisition in our State) presided, assisted by Dr. J. G. Harper, and by whom the manipulation of Barnum's Dam, Varney's pluggers, heavy *vs.* light mallets, was fully discussed, commented upon and shown to the members of the Association as well as others present.

The conventien adjourned at 3:30 p. m. on Thursday, to meet again on the last Wednesday of next May, 1875, in the city of St. Paul, when it is hoped to continue the increasing interest shown by those of the profession who have just closed up their annual convention at the beautiful town of Faribault.

